# The effect of Sancai powder on glycemic variability of type 2 diabetes in the elderly: A randomized controlled trial

**DOI:** 10.1097/MD.0000000000020750

**Published:** 2020-07-31

**Authors:** Dongqi Zhou, Li Zhang, Xuke Han, Yang Gao, Min Zeng, Weiwei Yu, Lisha Sun, Qiu Chen

**Affiliations:** a Hospital of Chengdu University of Traditional Chinese Medicine; b Chengdu University of Traditional Chinese Medicine, Chengdu, Sichuan Province, China.

**Keywords:** elderly patients, randomized controlled trial, Sancai powder, type 2 diabetes mellitus

## Abstract

**Background:**

Type 2 diabetes is a kind of metabolic disease. Its clinical characteristic is hyperglycemia. Recently, more and more elderly people suffer from type 2 diabetes, and the glycemic variability of the elderly is greater. In addition, blood sugar variation is more likely to cause diabetes complications than simple hyperglycemia. Sancai podwer (SC) is based on the theory of traditional Chinese medicine and gradually formed in the summary of clinical experience. It has the effect of lowering blood sugar and alleviating clinical symptoms of diabetes. But the existing evidence of its efficacy on glycemic variability is insufficient. So, in our study, the randomized controlled trials will be used as a research method to explore the effects of SC on glycemic variability of type 2 diabetes.

**Method:**

We will use randomized controlled experiments based on the recommended diagnostic criteria, inclusion and exclusion criteria. A total of 60 elderly patients with type 2 diabetes will be randomly divided into treatment group and control group, 30 cases in each group. The control group will receive conventional western medicine and the intervention group will receive SC combined with western medicine. The standard deviation and coefficient of variation of blood glucose level will be used as evaluation indexes.

**Discussion:**

This study can provide evidence for the clinical efficacy and safety of SC in elderly patients with type 2 diabetes mellitus.

**Trial registration:**

This study is registered on the Chinese Clinical Trial Registry: ChiCTR2000032611.

## 1 Introduction

Type 2 diabetes mellitus (T2DM) is a metabolic disease. Its clinical feature is hyperglycemia, and T2DM is also a common clinical endocrine disease and elderly chronic disease.^[1]^ With the aging of populations worldwide growth, T2DM is increasingly common in the elderly.^[2]^ T2DM occurred in 9% of the adult population, about 20% over the age of 65.^[3]^ According to the World Health Organisation, in 2019, it is estimated that diabetes over 65 years old is 111 million and will reach 276 million by 2045.^[4]^ Inevitably, this will cause further pressure on healthcare resources and medical service providers.^[5]^

Due to the course of type 2 diabetes in elderly patients is generally long and the condition is complex, the function of islet B cells is gradually exhausted, and the ability of the body to regulate blood glucose is relatively weak.^[6]^ The amplitude of blood glucose fluctuation in elderly patients increases accordingly.^[7]^ To make matters worse, unstable blood sugar is closely related to the occurrence and development of chronic complications of diabetes.^[8–11]^ Blood glucose fluctuations are significantly associated with the severity of coronary heart disease and the recurrence of acute myocardial infarction in diabetic patients.^[8,9]^ The glycemic variability is also a relevant risk factor for diabetic retinopathy.^[12]^ Long-term blood glucose fluctuation will affect the occurrence and development of chronic kidney disease.^[13–15]^

Western medicine's treatment of type 2 diabetes with oral medicine revolves around aspects such as promoting insulin secretion, improving insulin resistance, and delaying the absorption of glucose in the gastrointestinal tract. Although it can effectively lower blood sugar, the treatment target is relatively single and cannot effectively relieve oxidative stress and vascular endothelial damage. In addition, it has some side effects and adverse reactions. The common adverse reactions caused by the clinical use of hypoglycemic drugs mainly include hypoglycemic reactions, digestive tract reactions, allergies, and weight gain, severe may cause liver damage.^[16–18]^ Research data show that the incidence of adverse reactions of taking hypoglycemic drugs for type 2 diabetes is 7.6%.^[19]^

SC was founded on the basis of 3 ancient Chinese medicine prescriptions, namely ”San Cai Tang,” “Jiao Tai Wan,” and “Fructus Mume.” The reason for choosing these Chinese herbal medicines to treat diabetes is based on the theory of traditional Chinese medicine (TCM). SC can reduce blood sugar and there is a certain pharmacological research foundation (Table 1). Moreover, our team has conducted a single-blind randomized controlled trial and confirmed that SC can effectively improve endothelial dysfunction (ED), insulin resistance (IR), hyperinsulinemia, and mild vascular dementia (VaD) symptoms.^[20]^ The mechanism of IR is very complicated. So far, many basic researches and clinical trials have proved that IR is associated with inflammation, oxidative stress (OS), and abnormal signal pathways in cells.^[21]^ In addition, oxidative stress will aggravate the damage of islet function.^[22]^ Under the combined effect of the above mechanisms, the variability of blood glucose will also increase. The elderly are more susceptible to various unfavorable factors due to their long course of illness and complicated condition, leading to unstable blood glucose. Therefore, the emphasis of this study is to observe the safety and clinical effect of SC and Western Medicine on T2DM. We hope that through this study, we can find more evidence-based medical evidence that SC reduces blood glucose variability, and provide patients with more treatment methods.

**Table 1:**
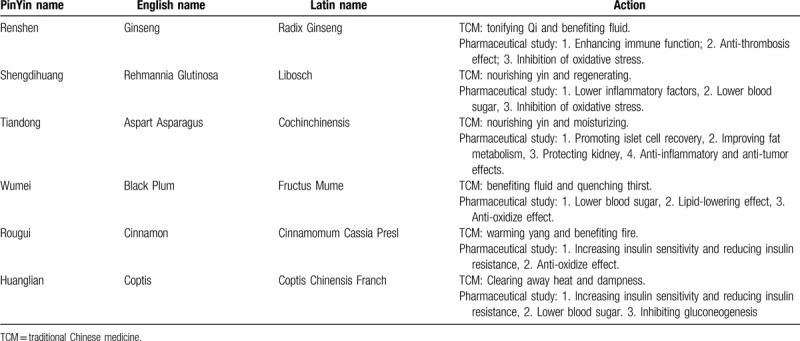
The action of each Traditional Chinese medicinal herb.

## 2 Methods/Design

### 2.1 Hypotheses

SC could reduce blood glucose variability in elderly patients with type 2 diabetes and relieve clinical symptoms.

### 2.2 Objectives

1. To compare the difference in blood glucose variability between the intervention group and the control group through standard deviation of blood glucose level (SDBG) and coefficient of variation (CV).
2. To compare the difference in the improvement of diabetes symptoms between the intervention group and the control group.
3. To analyze the synergy or other effects of SC on type 2 diabetes.

### 2.3 Study design and settings

**Figure 1:**
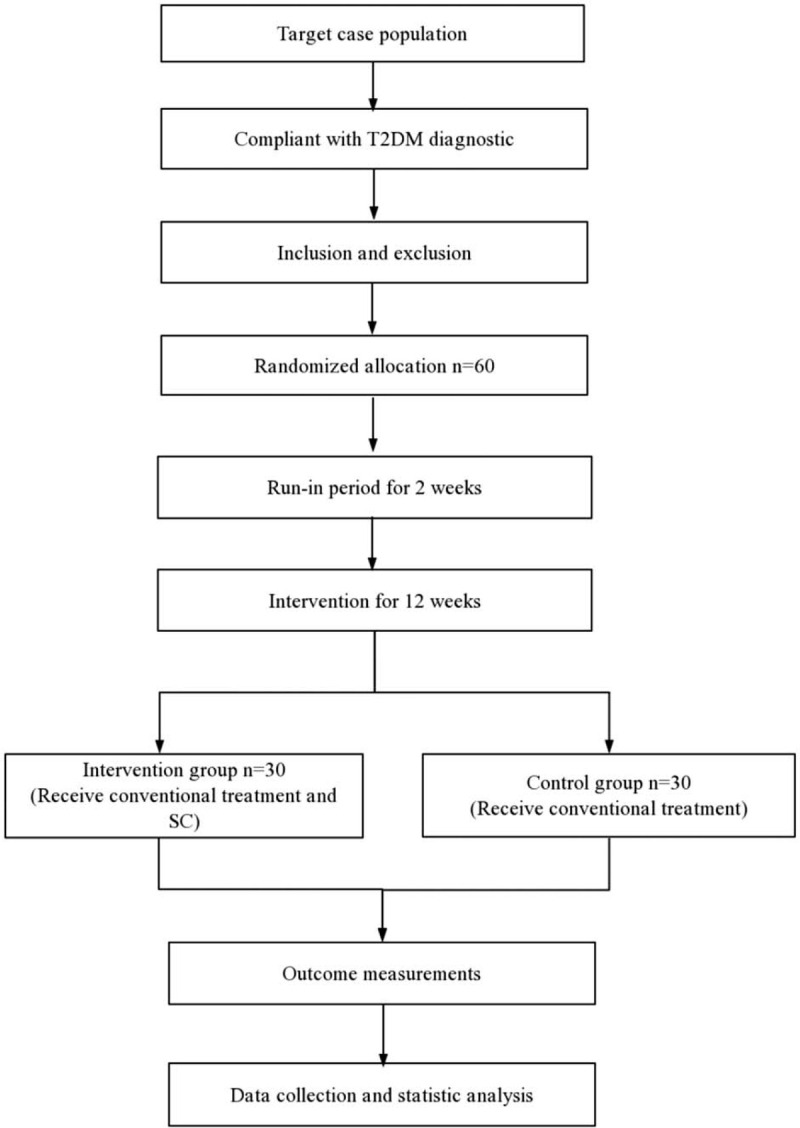
Flowchart of the trial. Figure shows the flowchart of the study process.

The study will be a double-blind, randomized controlled trial. It will be carried out in the Hospital of Chengdu University of Traditional Chinese Medicine. The study protocol follows the recommendations of the Standard Protocol Items for Randomized Trials and the Consolidated Standards of Reporting Trials Extension for CHM Formulas statemen.^[23,24]^ The author of this protocol will truthfully inform the participants of this experimental study, procedures, potential risks, and benefits. With their consent, they will sign an informed consent form and abid by the schedule represented in Fig. 1. It is worth mentioning that participants can only participate in the study if they have fully read, agreed, and signed an informed consent form.

### 2.4 Participants

Participants will be composed of outpatients and inpatients of the Hospital of Chengdu University of Traditional Chinese Medicine. T2DM diagnosis based on World Health Organization (1999) criteria.^[25]^

#### 2.4.1 Western medicine diagnostic criteria

(1) Islet cell antibody (ICA), glutamate decarboxylase antibody (GADA) are negative.
(2) Typical diabetes symptoms and random venous PG ≥ 11.1 mmol/L or FPG ≥ 7.0 mmol/L or in an oral glucose tolerance test, 2hPG ≥ 11.1 mmol/L, or have been diagnosed as diabetes in the past.

#### 2.4.2 TCM diagnostic criteria

Based on the relevant standards in “Guiding Principles of Clinical Research on New Drugs of Traditional Chinese Medicine (2002)” and “Guidelines for Prevention and Treatment of Diabetes TCM (2007),” the dialectical standards for the syndrome of yin deficiency and heat in this study were formulated. The symptoms can be seen: dry mouth and throat, polydipsia and polyuria, polyphagia, irritability, red face, night sweats, red tongue or crimson tongue, thin white tongue coating or thin yellow tongue coating or less tongue coating, thready and rapid pulse or taut and rapid pulse.

#### 2.4.3 Inclusion criteria

The research will be carried out in China. Patients will be enlisted from the Hospital of Chengdu University of Traditional Chinese Medicine. We will recruit participants according to the following inclusion criteria

(1) Participants are 65 to 85 years old.
(2) Meet the diagnostic criteria for type 2 diabetes.
(3) The BMI is between 18 and 28 kg/m^2^.
(4) Blood biochemical indicators such as liver and kidney function are normal.
(5) Accept and sign the informed consent.

#### 2.4.4 Exclusion criteria

Patients will be excluded if they meet the following criteria:

(1) Glycated hemoglobin is >10%.
(2) Diabetes is in the period of acute complications, such as diabetic ketoacidosis, diabetic hyperosmolar nonketotic coma, hypoglycemic coma, or severe unconscious hypoglycemia.
(3) Diabetic patients with severe chronic complications, such as diabetic foot with infection, diabetic nephropathy with renal insufficiency, diabetic retinopathy of >3 stages, etc.
(4) Patients with anxiety, depression, mania, or other mental illness.
(5) Patients with a history of stroke, transient ischemic attack, unstable angina, and myocardial infarction in the first 6 months of the study were patients with congestive heart failure.
(6) A history of acute or chronic pancreatitis.
(7) Allergic to Chinese herbal medicine.
(8) The history of the use of immunosuppressants and glucocorticoids in the past 3 months.
(9) Involuntary ability and other diseases affecting glucose metabolism.
(10) Patients who were enrolled in other clinical tests in the past 3 months.

### 2.5 Sample size calculation

The study used SBDG and CV as the main outcome indicators. In the preliminary experiment, the mean value of SBDG between the 2 groups was 0.39, and the standard deviations of treatment group and control group were 0.28 and 0.35 respectively. On the other hand, the mean value of CV between the 2 groups was 0.41, and the standard deviations of treatment group and control group were 0.31 and 0.67 respectively. Set *α* to 0.05 and *β* to 0.1. The sample sizes calculated using PASS 11 software were 14 and 25, respectively. In order to ensure statistically meaningful results, considering the 20% dropout rate, and according to the formula of n ^ = n/(1–f), the total number of studies was determined to be 60 cases, 30 cases per group.

### 2.6 Randomization and blinding

A software called SPSS Statistics Version 17.0 (IBM Corp., Armonk, New York) will be used to generate randomization sequence. The randomization sequence will be concealed and disseminated using opaque envelopes. Participants will be randomly divided into an intervention group and a control group in this way. In the progress of our study, evaluators, participants, and experimental researchers will be blinded. Unblinding is allowed to be performed only when the participant has an adverse reaction. The first time an adverse reaction occurs, the principal investigator will immediately assess the patient's condition and record in detail the time, place, and possible cause of the adverse reaction in Case Report Forms. In this experiment, the drug distribution managers and data analysis experts will not be directly involved in the process of the study and intentional analysis will be applied to the unblinding participants.

## 3 Interventions

### 3.1 Run-in period

According to the 2010 China Type 2 Diabetes Clinical Practice Guidelines,^[26]^ all participants will receive lifestyle interventions for 2 weeks, in order to standardize their diet and exercise therapy.

### 3.2 SC intervention

#### 3.2.1 Control group

As metformin is currently the first-line medication for T2DM, participants in this group will take 500 mg of metformin orally 3 times a day (if there is gastrointestinal discomfort, it can be taken during meals or 15 minutes after meals); the course of treatment is 3 months.

#### 3.2.2 Intervention group

Participants in this group will take the same dose of metformin as the control group. In addition, they will take SC. In this study, SC was provided by the Hospital of Chengdu University of traditional Chinese Medicine. The composition of the drug is: Ginseng, Rehmannia Glutinosa, Aspart Asparagus, Black Plum, Cinnamon, Coptis. The formula is made into powder according to the compatible dosage. Each dose is 88.5 g, divided into 3 parts, each part is taken with 200 mL of boiling water and taken 20 minutes before 3 meals. No other drugs are used during treatment period. It is worth mentioning that the change of conditions should be closely monitored at this time so as to control the deterioration of conditions in time.

## 4 Outcome measures

We will collect the characteristics of the 2 groups of participants at baseline and after the intervention, such as age, sex, physical examination, biochemical indicators, and other data (twice in total). The primary outcome indicators and secondary outcome indicators are measured and calculated at baseline, the first month, the second month, and the third month, respectively. (Four times in total.)

### 4.1 Primary outcome measures

We will use the SDBG and CV as the main outcome measures.

### 4.2 Secondary outcome measures

We will use mean blood glucose (MBG), largest amplitude of glycemic excursions (LAGE), postprandial glycemic excursions (PPGE), glycated hemoglobin as the secondary outcome measures. In the whole process, we will also collect the following indicators:

(1) Total effective rate; the judgement of effectiveness takes into account the improvement of examination indicators and the relief of clinical symptoms.
(2) Hyperglycemia or hypoglycemia that may occur during the study.
(3) Have any adverse effects of medication throughout the process.

### 4.3 Efficacy evaluation

By calculating the SDBG and CV of the participants at baseline, the first month, the second month, and the third month, the efficacy will be evaluated (Table 2). At the same time, we will also evaluate MBG, LAGE, and PPGE, glycated hemoglobin as secondary efficacy evaluations. We will use a simple method is to get blood sugar and calculate the above indicators which is finger-prick measurements within a specific period, 7-point profiles (7 am, 9 am, 11 am, 1 pm, 5 pm, 7 pm, and 9 pm). Despite the lack of nocturnal blood glucose measurements, the SDBG can be an appropriate data even in data sets in which glucose values do not follow a Gaussian distribution, because it has a near linear relation with the interquartile range.^[27,28]^ In addition, we will measure the following indicators at baseline and at the end of the experiment: blood pressure, fasting plasma glucose, total cholesterol, low density lipoprotein cholesterol, high density lipoprotein cholesterol, triglycerides, advanced glycation end products, and changes in the scores of TCM syndromes measured by questionnaires.

**Table 2:**
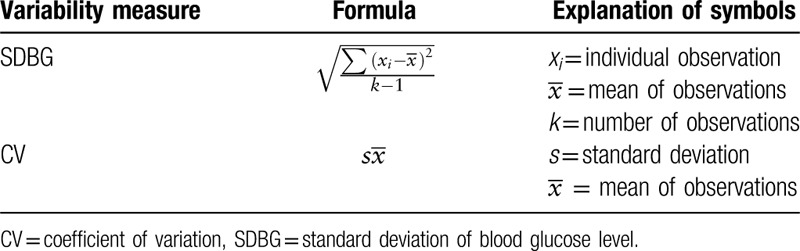
The calculation formulas.

### 4.4 Conditions for suspension and withdrawal of clinical trials

The research staff of this clinical trial should record in detail the reasons why the participant proposed to withdraw from the clinical trial or the subjects’ reluctance to continue the clinical trial, and record in detail the evaluation indicators at the time of drug withdrawal. In addition, the reason for discontinuing the trial and its relationship with the clinical trial should be clearly documented. When the participants have the following conditions, we will suspend and withdraw clinical trials.

(1) People who quit the study themselves.
(2) People who can’t insist on treatment.
(3) In case of allergic reaction or serious adverse reaction during the test, the test shall be suspended. The ethics committee will assess any correlation between the adverse event and the intervention, and make a decision on whether the study should proceed.
(4) Those who did not follow the plan strictly.
(5) People who joined other clinical trials in the course of this experiment.

### 4.5 Statistical analysis

Data processing uses SPSS statistical software version 17.0 (IBM Corporation). Normally distributed variables are expressed as mean and standard deviation; non-normally distributed variables are expressed as median and quartile range. Normally distributed variables between groups using independent sample *t* test, rather than the normal difference variables using Mann–Whitney *U* test. In the comparison group, paired *t* test variables were normally distributed, non-normally distributed variables the Wilcoxon rank sum test. *P* < .05 was considered a statistically significant difference. Study figures were created using SigmaPlot (Systat Software Inc.).

### 4.6 Monitoring

According to recommendation of the National Institutes of Health (NIH), this trial established an independent Data and Safety Monitoring Board (DSMB). The committee consists of 3 members, including a Chinese medicine clinician, an endocrinologist, and a statistician. The committee will supervises whether the study follows the protocol design and standard guidelines, will monitor the progress of the trial, and will observe whether adverse events and etiologies are adequately recorded. This DSMB also identify problems in the project, if any; the committee will make the decisions to change the details of this protocol and announce the research personnel by written notice after approval by the ethics committee. Moreover, a qualified clinical trial expert will be invited to monitor this trial and the will take full responsibility and make the final decision.

### 4.7 Data management

We will prepare a paper version of the Case Report Form for each participant in advance. After collecting the participant information, the researcher will fill it out manually. At the same time, the participant's information will be input into the Excel file for later statistical analysis. We will truthfully inform the above information processing methods and execute after obtaining the consent of the participants. The personal information of each participant will be kept strictly confidential. In addition, the consent form signed by the participants in advance will also be kept in a file bag and sealed. All data will be available from the 3rd month to the 3rd year after completion and release. For reasonable data requirements, please contact the corresponding author.

### 4.8 Ethics

This study was designed in accordance with the Declaration of Helsinki and the study protocol was approved by the Ethical Review Committee of Hospital of Chengdu University of Traditional Chinese Medicine (Chengdu, China). Each participant will voluntarily participate in the trial and sign informed consent.

## 5 Discussion

Elderly patients with type 2 diabetes are more prone to hypoglycemia and blood glucose variation. In terms of physiology, with increasing age, the plasma albumin concentration of the elderly gradually decreases, and the drug–protein binding rate decreases, resulting in an increase in free drug concentration and an increase in the effect of the drug. At the same time, the decrease in liver blood flow and the number of functional hepatocytes and the decrease in the activity of liver microsomal enzymes have led to a decline in the liver's ability to metabolize drugs in the elderly and a prolonged plasma half-life. Renal function declines with age, glomerular filtration rate decreases, renal blood flow decreases significantly, and renal tubular function declines. These changes easily make the elderly more likely to cause hypoglycemic reactions when using conventional doses of hypoglycemic drugs, thus making the elderly more variable in blood glucose.^[29,30]^ In terms of pathology, with the progress of the disease, decreased islet function reduces the ability to regulate blood sugar. The vulnerability of the elderly to oxidative stress, inflammatory factors, and endothelial dysfunction makes the islet resistance even worse. Under the influence of these factors, the variability of blood sugar increases, making the risk of diabetes complications in elderly patients increased. Glycemic variability is positively associated with micro- and macrovascular complications, which have more deleterious effects than sustained hyperglycemia in the pathogenesis of diabetic cardiovascular complications. Glycemic variability causes vascular injury by increasing oxidative stress and endothelial dysfunction, and exacerbating chronic inflammatory state.^[31,32]^ What can we do to reduce the variability of blood glucose in elderly patients with type 2 diabetes and find an effectual and acceptable method has become one of the increasing concerns in the medical field. TCM has the advantages of safe use, less adverse reactions, and side effects, in addition, it also has a mature overall concept and a large number of clinical experience summary. From the perspective of dialectical holistic view, traditional Chinese medicine regulates the balance of yin and yang in the human body, and has unique advantages such as flexible composition compatibility and personalized dialectical treatment. Chinese medicine has accumulated in long-term clinical practice, a wealth of experience in the diagnosis and treatment, but this is a subjective evaluation, is considered to be low-quality evidence based medicine. Therefore, it is important to conduct well-designed clinical studies to provide high-quality EBM evidence. The test is valuable because it can provide scientific evidence and rigorous EBM efficacy and safety about SC on glycemic variability of type 2 diabetes in the elderly.

## 6 Trial status

Recruitment of trial participants will begin in September 2020 and collection of trial data will continue until the end of June 2021.

## Acknowledgments

The authors would like to thank all the participants in this study. In addition, the authors express their most sincere gratitude to the hospital staff, the nurse, trial coordination group, and the research department for this study.

## Author contributions

**Conceptualization:** Dongqi Zhou, Li Zhang, Xuke Han, Yang Gao, Qiu Chen.

**Data curation:** Dongqi Zhou, Li Zhang, Yang Gao, Qiu Chen.

**Funding acquisition:** Qiu Chen.

**Investigation:** Xuke Han, Weiwei Yu.

**Methodology:** Dongqi Zhou, Weiwei Yu, Min Zeng.

**Project administration:** Qiu Chen.

**Resources:** Dongqi Zhou, Yang Gao, Qiu Chen.

**Supervision:** Yang Gao, Qiu Chen.

**Writing – original draft:** Dongqi Zhou, Li Zhang.

**Writing – review & editing:** Qiu Chen, Lisha Sun.
